# Correlation of hK6 expression with tumor recurrence and prognosis in advanced gastric cancer

**DOI:** 10.1186/1746-1596-8-62

**Published:** 2013-04-15

**Authors:** Xunqi Liu, Hailin Xiong, Jun Li, Ying He, Xia Yuan

**Affiliations:** 1 Sun Yat-sen university, Guangzhou City, Guangdong province, China; 2 Department of Oncology, HuiZhou Municipal Central Hospital, Huizhou City, Guangdong Province, China; 3 Department of Oncology, HuiZhou Municipal Central Hospital, Huizhou, Guangdong Province, 516001, China

**Keywords:** Stomach neoplasms, Human kallikrein-related peptidase 6 (hK6), Recurrence, Tumor markers

## Abstract

**Background:**

Human kallikrein gene 6 (KLK6) is a member of the human kallikrein gene family (Kallikreins, KLKs). Human kallikrein-related peptidase 6 (hK6) is a trypsin-like serine protease encoded by the KLK6, has been reported to be highly expressed in several cancers including gastric cancer. In this study, we investigated the the correlation of hK6 expression with clinicopathological characteristics, tumor recurrence and prognosis in advanced gastric carcinoma after curative resection.

**Methods:**

We retrospectively analyzed the clinical data of 129 cases advanced gastric cancer after curative gastrectomy. The expression of hK6 in advanced gastric cancer tissues compared to adjacent noncancerous tissues were examined, and the relationship between hK6 expression and clinicopathological characteristics was evaluated. In additional, these patients were followed up to investigate the relationship between hK6 expression and the survival time.

**Results:**

The positive rate of hK6 expression was significantly higher in advanced gastric cancer tissue, than that in adjacent noncancerous and gastric ulcer tissues (36.5%, 33.3%, respectively, P < 0.001). There was a close relationship between hK6 expression and TNM stage (P = 0.005), vascular invasion (P = 0.037) and perineural invasion (P = 0.035). Furthermore, patients with hK6 positive showed significantly higher recurrence and poorer prognosis than those with hK6 negative. Multivariate analysis showed that hK6 expression was a significant independent factor for tumor recurrence and overall survival.

**Conclusion:**

hK6 is overexpressed in advanced gastric cancer tissues. Its clinical utility may be used as an unfavorable indicator in predicting tumor recurrence and prognosis for advanced gastric cancer after operation. This study also suggests that hK6 might be a potential therapeutic target for gastric cancer.

**Virtual slides:**

The virtual slide(s) for this article can be found here: http://www.diagnosticpathology.diagnomx.eu/vs/8558403578787206

## Introduction

Gastric cancer is one the most common malignancies and the second leading cause of death among all cancers in the clinical [1], and surgical resection remains currently the only treatment with curative. Even if the surgery, postoperative chemo-radiotherapy immunotherapy, targeted therapy those multidisciplinary collaborative mode have improved the survival rate of gastric cancer patients. Still the postoperative recurrence rate of gastric cancer is high, especially advanced gastric cancer (Advanced gastric cancer refers to that tumor invades to the muscularis propria or through the muscularis to serosa and extra serosa). And early diagnosis is difficult, the specific tumor markers in detection of tumor recurrence has not yet been discovered.

The KLKs consist of 15 homologous genes which encoding secreted serine proteases, localized in tandem on chromosome 19q13.4. The KLKs have similar genomic organizations, and show significant homology at both the nucleotide and protein level. The same gene of the family is expressed in different tissues, and multiple genes are commonly expressed in the same tissue [2].

KLK6 is a member of the KLKs, which encodes for human kallikrein-related peptidase 6 (hK6). It is a serine protease made up of 223 amino acids with trypsin-like activity. hK6 has been cloned independently by three groups, using a differential display technique from primary and metastatic breast cancer cell lines. Anisowicz et al. [3] first isolated the full-length cDNA of KLK6, named protease M, which is strongly expressed at the mRNA level in certain primary breast cancer cell lines and in ovarian cancer tissues and cell lines. Yamashiro et al. [4] cloned this same gene, named neurosin, from a cDNA library prepared from a human colorectal cancer cell line (COLO 201). Finally, Little et al. [5] cloned the identical cDNA, named zyme, from the brain tissue of a patient with Alzheimer’s disease, which played an important role in the development and progression of Alzheimer’s disease. Study found that hK6 was highly expressed in gastric cancer and indicated poor prognosis [6].

To our best knowledge, few studies have been investigated concerning the clinical significance of hK6 expression in advanced gastric cancer with recurrence and prognosis. In this study, we evaluated the expression of hK6 in the surgical specimens of advanced gastric cancer tissues, paired adjacent noncancerous tissues and gastric ulcer tissues using immunohistochemistry HRP, and analyzed their correlations with clinicopathological characteristics and patients survival to clarify the significance of hK6 in advanced gastric cancer after curative surgery.

## Materials and methods

### Patients and collection of tissue samples

A total of 129 samples were obtained from patients from January 2007 to November 2011 who underwent curative surgery for advanced gastric cancer in HuiZhou Municipal Central Hospital of GuangDong. Resection samples were confirmed to be primary advanced gastric cancer by clinical pathology, none of the patients received any chemotherapy, radiotherapy and other adjuvant therapy prior to operation. 52 blocks from adjacent noncancerous gastric tissues (at least 5 cm away from the cancer margin) were obtained from the patients. In addition, 36 specimens obtained from patients who underwent surgery and were confirmed to be gastric ulcer used as controls in the same period.

Patients^,^ clinicopathological characteristics such as gender and age, tumor location, size, differentiation, T stage, lymph node metastasis, TNM stage and whether recurrence were retrospectively reviewed. The mean age was 60 years (range: 28 ~ 80 years)with 56 women and 73 men. All the patients were staged based on the TNM classification of the American Joint Committee on Cancer (AJCC, 7th Edition criteria, 2010) [7].Tumor location in gastric fundus/cardia was 21 cases, gastric body 32 cases, gastric antrum 76 cases. Tumor size was greater than 5 cm in 70 cases, and less than 5 cm in 59 cases, 16 cases classified as well or moderately differentiated(including tubular and papillary adenocarcinoma), 113 cases as poorly differentiated (poorly differentiated adenocarcinoma and other cell types such as mucinous adenocarcinoma and signet ring cell carcinoma and so on), 19 cases were at T2, 55 cases were at T3 and T4 respectively. N0/N1 was found in 49 cases, N2/N3 in 80 cases, 43 cases withI/II stage, 86 cases with III/IV stage, written informed consent was obtained from all patients or their families. The study was approved by the Institute^,^s Ethics Committee of HuiZhou Municipal Central Hospital. All the specimens were fixed in 10% neutralized maldehyde, embedded in paraffin, cut into 3-μm thick sections, and mounted on glass slides for immunohistochemistry analysis.

### Immunohistochemistry

Immunohistochemistry was performed using the Horseradish Peroxidase two-step method, which avoids the interference of endogenous biotin, thus reducing the nonspecific false-positive background staining. Immunohistochemistry was carried out in eight steps according to the instructions: 1, The paraffin sections were deparaffinized in xylene, rehydrated through gradient ethanol. 2, Antigen retrieval was performed in Tris-EDTA buffer (pH 9.0) with a pressure cooker. 3, Block the endogenous peroxidase with 3% hydrogen peroxide. 4, Then incubated with the purified rabbit polyclonal antibody(AP6325a, Abgent, San Diego, USA), antigen retrieval and dilution were determined by preliminary experiments. 5, Detection of hK6 immunocomplex was incubated with the PV9000 kits (Golden bridge Biotechnology Co, Ltd, Beijing, China). 6, Stained with diaminobenzidine substrate chromogen solution (Goldenbridge Biotechnology Co, Ltd) until the optimal staining was achieved. 7, The sections were counterstained with hematoxylin, differentiated, returned blue. 8, Finally, dehydrated through gradient alcohol, cleared in xylene, neutral gum mounted. Then observed under the optical microscope.

### Immunohistochemical staining score

All slides were assessed separately by two senior pathologists with no prior knowledge of clinicopathological parameters, the final score for their average. For evaluation of hK6 immunohistochemical staining, the percentage of positive cells was evaluated quantitatively 10 fields within the tumor were selected randomly, expression in 1000 cancer cells (100 cells per field) was evaluated using a high-power magnifcation (400×) and an average was evaluated quantitatively and scored as: 0 for the percentage of positive cells ≤ 10%, 1 for staining of 11 ~ 25%, 2 for staining of 26 ~ 50%, and 3 for staining of 51 ~ 75%, 4 for staining of > 75% of the cells examined [6], the staining intensity was respectively graded semiquantitatively as follows: 0 no signal, 1 weak, 2 moderate, and 3 strong according to the previous literatures [8], a total staining score of 4 or more was graded as immunohistochemical positive.

### Follow-up

All these patients received fluorouracil based adjuvant chemotherapy after surgery. As a regular follow-up, all the patients were checked every 3 months during the first 2 years and every 6 months during the third to the fifth year, once a year thereafter. Medical work-up consisted of history and physical examination, hematology and biochemical tests including serum CEA, CA19-9 and CA72-4, and imaging including chest radiography, barium meal, abdominal ultrasonography (US), computed tomography (CT), and endoscopy according to the clinical situation. The follow-up time had the day of surgery as a starting point, the time of tumor recurrence and death were recorded, these two points were evaluated for prognostic analysis. If the follow-ups were incomplete, patients or their families were contacted by telephone, the closing date for follow-up was May 2012, the median follow-up period after surgery was 25 months (range 4–64 months).

### Statistical analysis

Statistical analysis was carried out using the SPSS 13.0, the relationships between hK6 expression and patient clinicopathological characteristics were analyzed with the Chi-square (χ^2^) and Fisher’s exact test. For survival analysis, two end points were examined:recurrence-free survival (RFS) and overall survival (OS) respectively. RFS is defined as the time interval between the date of surgery and the date of first detected recurrence or metastasis, and OS is defined as the time interval between the date of surgery and the date of death or the date of last follow-up for those who were alive at the end of the study. The Kaplan–Meier model was used to examine survival between the patients positive and negative hK6 expression. The significance of any difference in the survival curves was determined with the log-rank test. Variables achieving a significance level in the univariate analysis were subsequently introduced in a forward LR proportional hazard analysis (Cox’s model)to identify those variables independently associated with survival, the p value of 0.05 (or less)was considered as statistically significant.

## Results

### hK6 expression in advanced gastric cancer tissues

**Figure 1:**
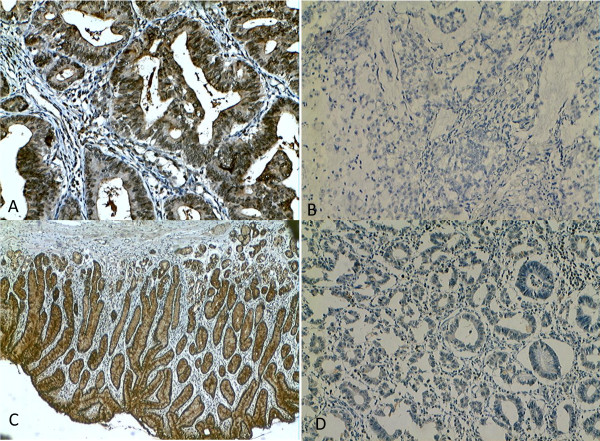
**Immunohistochemical staining of hK6 in the advanced gastric carcinoma (A, B) and adjacent noncancerous (C, D) tissues. A** hK6 positive expression; **B** hK6 negative expression; **C** hK6 positive expression; **D** hK6 negative expression.

Different proteins were expressed in different location of the cell, such as cytoplasm, membrane, nuclear [9,10].hK6 positive staining was mainly expressed in the cytoplasm of gastric cancer cells as brown or dark brown granules (Figure 1) also was observed in the adjacent noncancerous and gastric ulcer epithelial cells by immunohistochemistry. The positive rate of KLK6 expression in gastric cancer tissues was 77.5% (100/129), significantly higher than that in adjacent noncancerous tissues (36.5%) and ulcer (33.3%) tissues respectively (P <0.001; Table 1).

**Table 1:** The expression of hK6 in adjacent noncancerous, gastric ulcer and advanced gastric cancer tissues

**Group**	**Cases**	**Positive**	**Positive rate**	**χ**^**2**^**-value**	**P-value**
GCT	129	100	77.5%	39.201	<0.001^a^
ANT	52	19	36.5%	0.096	0.757^b^
GUT	36	12	33.3%		

Abbreviation: *ANT*, Adjacent noncancerous tissues; *GUT*, Gastric ulcer tissues; GCT gastric cancer when GCT compared to ANT and GUT, P < 0.001, ANT compared to GUT, P = 0.757.

### hK6 expression and clinicopathological characteristics

The correlation between hK6 expression levels and clinicopathological characteristics is as summarized in Table 2, hK6 expression significantly correlated with TNM stage (P = 0.005), vascular invasion (P = 0.037) and perineural invasion (P = 0.035). hK6 positive tumors more frequently had later stage, an increased incidence of vascular and perineural invasion, on the other hand, no statistical significant difference was observed between its expression and gender, age, tumor site, size, differentiation, T stage, lymph lodes metastasis or margin (P > 0.05).

**Table 2:** The relationship between KLK6 expression, tumor recurrence and clinicopathological characteristics

**Characteristics**	**Cases**	**Positive**	**Positive rate**	**χ**^**2**^**-value**	**P-value**
Gender				1.214	0.271
Female	56	46	82.1%		
Male	73	54	74.0%		
Age				0.067	0.796
<60 years	65	51	78.5%		
≥60 years	64	49	76.6%		
Tumor site				3.806	0.149
fundus/cardia	21	19	90.5%		
body	32	26	81.3%		
antrum pylorus	76	55	72.4%		
Tumor size				0.540	0.462
<5 cm	59	44	74.6%		
≥5 cm	70	56	80.0%		
Differentiation				0.004	0.951
Well/moderate differentiated 16	16	13	81.3%		
Poorly differentiated	113	87	77.0%		
T stage				1.486	0.476
T2	19	13	68.4%		
T3	55	45	81.8%		
T4	55	42	76.4%		
Lymph node				1.682	0.195
N0/N1	49	35	71.4%		
N2/N3	80	65	81.3%		
Vascular invasion				4.370	0.037
Absent	81	58	71.6%		
Present	48	42	87.5%		
Perineural invasion				4.439	0.035
Absent	76	54	71.1%		
Present	53	46	86.8%		
Margin				0.211	0.646
Absent	110	84	76.4%		
Present	19	16	84.2%		
TNM stage				8.029	0.005
I + II	43	27	62.8%		
III + IV	86	73	84.9%		

### hK6 expression association with tumor recurrence and poor overall survival

As the follow-up ended, 68 (52.7%) patients had died, A total of 61 patients died because of tumor progression and 7 due to other causes. In all, there were 69 (53.5%) patients developed tumor recurrence, 31 of whom developed as loco-regional relapse, 19 as distant metastases (11 in liver, 3 in lung, and 5 in bone), 14 as peritoneal recurrence and 5 as remnant; All but eight patients with tumor recurrence died from it.

**Figure 2:**
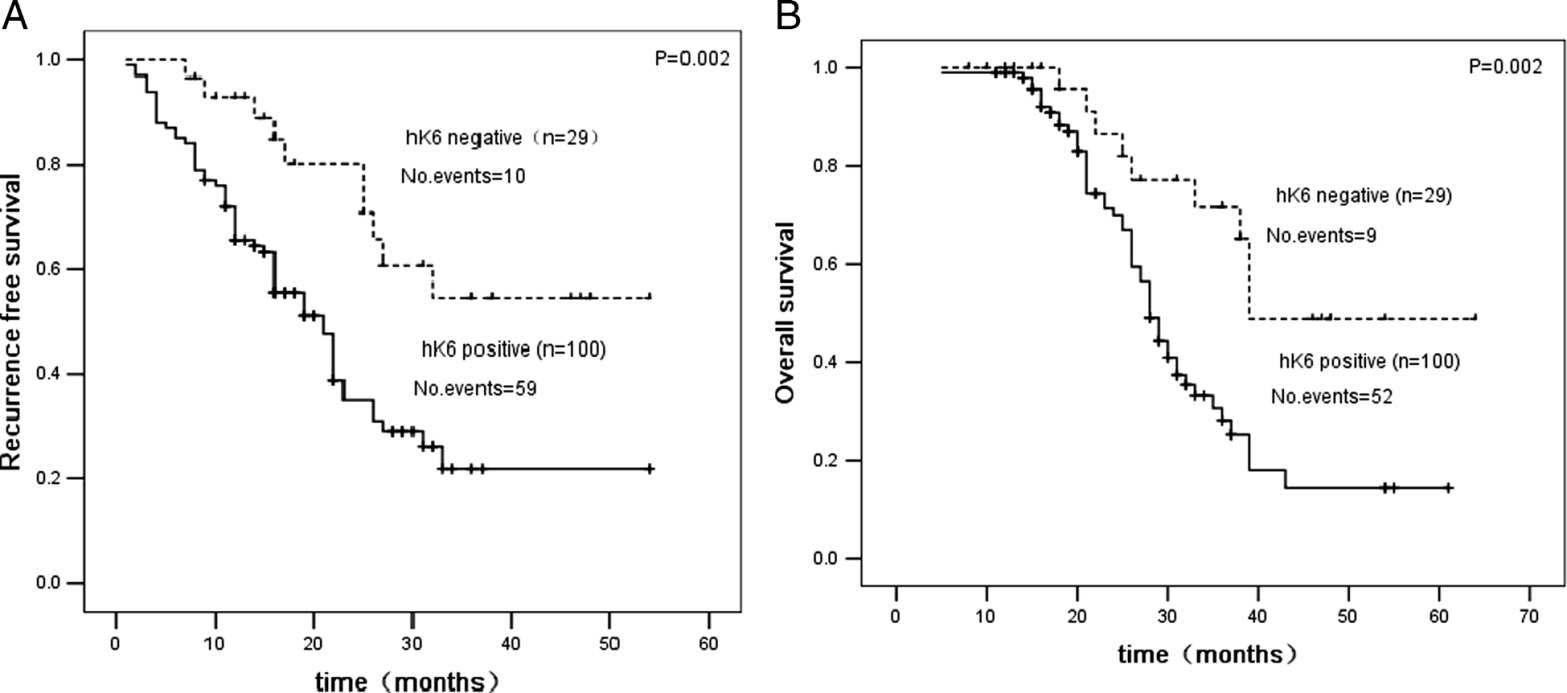
**Kaplan–Meier survival curves shows the probability of being free of recurrence (A) and overall survival (B) in patients with hK6-negative and hK6-positive expression.** Abbreviations: 95% CI 95% confidence interval, HR hazard ratio, OS overall survival, RFS recurrence-free survival.

Kaplan–Meier survival curves indicated patients with positive hK6 expression were more likely to have both a shorter RFS (P = 0.002, Figure 2A) compared to patients with negative hK6 expression. Univariate analysis expression with regard to RFS, using the Cox proportional hazard regression model, showed that hK6 expression, TNM stage, tumor size, T stage, lymph lodes, margin and were influencing factors for RFS, When hK6 expression along with other factors were examined in a multivariate model, TNM stage, hK6 expression, tumor size, margin were independent predictors of tumor recurrence (Table 3), indicating hK6 positive patients are twice more likely to have a recurrence than hK6 negative ones.

**Table 3:** Univariate and multivariate analysis of hK6 and other clinicopathological characteristics with regard to RFS in advanced gastric cancer

	**Univariate**			**Multivariate**		
**Variable**	**HR**	**95% CI**	**P**	**HR**	**95% CI**	**P**
Gender F/ M	1.05	0.65 -1 .69	0.86	–	–	–
Age < 60/ ≥60 yr	1.30	0.81- 2.08	0.29	–	–	–
Tumor site	–	–	0.58	–	–	–
body/fundus	0.68	0.31-1.49	0.33	–	–	–
antrum/fundus, body	0.88	0.45 -1.71	0.70	–	–	–
Tumor size < 5/ ≥5 cm	2.05	1.25 - 3.37	0.005	1.80	1.07 – 3.04	0.027
grade Well/Poorly	0.88	0.40 -1.93	0.75	–	–	–
T stage	–	–	0.10	–	–	–
T3/T2	2.21	0.92-5.30	0.077	–	–	0.58
T4/T2,T3	2.60	1.08 -6.26	0.032	–	–	0.75
N stage N0,N1/N2,N3	2.28	1.34-3.87	0.002	–	–	0.83
Vascular−/+	0.91	0.49-1.70	0.77	–	–	–
Perineural−/+	1.27	0.71-2.25	0.42	–	–	–
Margin−/+	2.42	1.41-4.15	0.001	2.49	1.44– 4.31	0.001
TNM stage I,II/III,IV	3.10	1.69-5.68	0.000	2.25	1.18 –4.30	0.014
hK6−/+	2.77	1.41-5.47	0.003	2.09	1.04 –4.	230.039

According to the survival analysis, Kaplan–Meier survival analysis showed that patients with positive hK6 expression have lower OS (P = 0.002, Figure 2B), univariate analysis revealed that positive hK6 expression, TNM stage, tumor size, lymph node, T stage, and margin are important factors influencing the OS rates, when these factors were involved in a multivariate analysis (Table 4), TNM stage and hK6 were independent prognostic factors of OS.

**Table 4:** Univariate and multivariate analysis of prognostic factors of OS in advanced gastric cancer

	**Univariate**			**Multivariate**		
**Variable**	**HR**	**95% CI**	**P**	**HR**	**95% CI**	**P**
Gender F/M	1.35	0.80-2.25	0.26	–	–	–
Age < 60/≥60 yr	1.59	0.95-2.67	0.08	–	–	–
Tumor site	–	–	0.17	–	–	–
body/fundus	0.47	0.20-1.09	0.078	–	–	–
antrum/fundus, body	0.57	0.29 -1.13	0.11	–	–	–
Tumor size < 5/ ≥5 cm	1.72	1.02-2.90	0.043	–	–	0.57
grade Well/Poorly	0.57	0.26-1.27	0.17	–	–	–
T stage	–	–	0.004	–	–	0.019
T3/T2	1.60	0.66-3.89	0.30	0.83	0.33–2.12	0.70
T4/T2,T3	3.36	1.38–8.15	0.007	1.86	0.73–4.70	0.19
N stage N0,N1/N2,N3	2.87	1.57–5.21	0.001	–	–	0.12
Vascular−/+	1.23	0.65-2.34	0.52	–	–	–
Perineural−/+	1.32	0.71-2.43	0.38	–	–	–
Margin−/+	1.29	0.72-2.31	0.40	–	–	–
TNM stage I,II/III,IV	4.05	2.04– 8.03	<0.001	2.99	1.45–6.16	0.003
hK6−/+	2.88	1.40-5.92	0.004	2.66	1.25–5.63	0.011

Multivariate analysis indicated that hK6 expression was one of the independent prognostic factors of overall survival for the patients with gastric cancer next to TNM stage (P = 0.011).

## Discussion

Studies found that hK6 was secreted to the extracellular matrix and involved in the degradation of matrix proteins including fibronectin, laminin, vitronectin, collagen and induced E-cadherin ectodomain shedding, reduced the cell-cell adhesion [11,12] and led to keratinocyte to promote tumor proliferation, migration, invasion and metastasis [13]. It is known that human hK6 is are involved in several malignancies, Several scholars have reported that hK6 is overexpressed in ovarian cancer [14], uterine serous papillary cancer [15], colorectal cancer [16], gastric cancer [6] and esophageal cancer [17], but underexpressed in salivary gland tumors [18] and renal cell carcinoma [19], which depends on the tumor tissue types and microenvironment [20]. hK6 promotes the growth of most tumors. However, when hK6 was overexpressed in breast cancer MDA-MB-231 cells, cells were resulted in marked reversal of their malignant phenotype, manifested by lower proliferation rates and saturation density, marked inhibition of anchorage independent growth, reduced cell motility and ability to form tumors. The mechanism may be that hK6 play a protective role against tumor progression mediated by inhibition of epithelial-to-mesenchymal transition [21].

To date, it has been shown that KLK6 is upregulated in such gastrointestinal malignancies as pancreatic and colon cancers [22]. Ogawa et al. [16] reported that KLK6 mRNA expression was significantly higher in cancerous than in corresponding noncancerous colorectal tissue. Kim et al. [23] examined the KLK6 mRNA and its corresponding protein levels using RT-PCR and ELISA, found that KLK6 mRNA was upregulated by about 8-fold, compared to nontumor regions, and serum hK6 levels from gastric cancer patients was 1.7-fold to healthy individuals. When inactivating KLK6 with small interference RNA(siRNA), cellular invasiveness decreased by 45%. He also discovered that KLK6 degrade extracellular matrix proteins to tumor invasiveness and metastasis. Exogenous overexpression of KLK6 led to decreased activity of the E-cadherin promoter. Moreover, our study showed the positive rate of hK6 expression in advanced gastric cancer tissue was significantly higher than that in adjacent noncancerous and gastric ulcer tissue. However, no difference was found between adjacent noncancerous tissue and gastric ulcer tissue. These findings suggest that hK6 expression may play an important role in gastric cancer development.

Ogawa et al. [16] reported that high KLK6 mRNA expression levels in colorectal cancer tissues were correlated with serosal invasion, liver metastasis, advanced Duke’s stage, However, limited reports suggested the relationship between KLK6 expression and clinicopathological characteristics. Nagahara et al. [6] reported that the overexpression of KLK6 was significantly associated with TNM stage, lymphatic invasion for patients with gastric cancer. In the current study, for the TNM stage, the positive rate of hK6 expression was significantly higher in later stage (stage III and stage IV: 84.9%) than that in earlier stage (stage I and stag II: 62.8%). The difference was statistically significant, demonstrating that hK6 positive expression was strongly correlated with TNM stage in advanced gastric cancer. Our study also showed that there was a relationship between hK6 positive expression with vascular invasion, which was consistent with Nagahara’s [6] report. Additionally, It was reported that hK6 positive expression was associated with perineural invasion, which not studied in Nagahara’s [6] report, but it needs further study to verify. We found no relationship between hk6 expression in advanced gastric cancer and the patient gender,age,tumor site, size, differentiation, T stage, lymph lode metastasis, which was in agreement with previous results [6].

A plenty of evidence has demonstrated that several members of the KLKs are potential cancer biomarkers [24]. The most widely known and used KLKs is KLK3. KLK3 gene encodes prostate-specific antigen (hK3), has been approved by the US FDA as the specific marker of prostate cancer, which is widely used for screening, diagnosing, determining the efficacy and assessing the prognosis of prostate cancer [25]. KLK2 protein (hK2), is also considered as a secondary marker of prostate cancer. Recent reports have indicated that hK6 high expression in ovarian cancer [26], colon cancer [16,27], pancreatic cancer [28], non-small cell lung cancer [29] and intracranial tumors [30] could be an independent prognostic factor, suggesting that hK6 may be a prognostic marker for these tumors.

Studies have suggested that high KLK6 expression was closely associated with shorter overall and recurrence-free survival for ovarian cancer [31] and colon cancer patients [32]. The same result was confirmed in advanced gastric cancer in our study. All of these suggest that KLK6 may be a potential biomarker in predicting tumor recurrence and therapeutic target for them. Many studies consistently showed that TNM stage was the most reliable prognostic indicator of gastrointestinal neoplasms such as gastric carcinoma, gastrointestinal lymphomas patients [9,10,33,34], while other characteristics, there was no agreement. Jin et al. [33] found Ezrin overexpression increased significantly in lymph node metastasis, Demirag et al. [10] showed PKP3 expression and poor prognosis appeared to correlate with lymph node number. While in the present study, we did not find any association between lymph node metastasis and prognosis in multivariate analysis.

With the development of molecular and proteomics in gastric cancer, many proteins have been the potential markers for early diagnosis, prognosis and therapeutic targets of gastric carcinoma, among them c-Met [9], PKP2 and PKP3 [10], Ezrin [33], are the known ones.Moreover, other molecular biomarkers are currently in study.

Few previous studies investigated the clinicopathologic significance of hK6 expression in advanced gastric cancer tissues. Thus, in this study, we examined the correlation of hK6 expression with tumor recurrence and prognosis in advanced gastric cancer. The findings indicate that the expression of hK6 was significantly associated with TNM stage, vascular invasion, perineural invasion and poor prognosis for patients with advanced gastric cancer. Moreover, we found that patients with positive hK6 expression were more likely to have recurrence, and lower RFS and OS rates compared with those with negative hK6 expression. Multivariate analysis proved that hK6 expression was a significant independent prognostic factor of RFS and OS in patients with advanced gastric cancer. Our findings support that hK6 might be used as a potential biomarker and therapeutic target for gastric cancer. Furthermore, positive expression of hK6 in advanced gastric cancer tissues may provide valuable information to understand the biological behavior of gastric cancer.

In conclusion, our study showed that hK6 expression in advanced gastric cancer significantly correlated with TNM stage, vascular invasion and perineural invasion as well as RFS and OS rates. This is a rare report to evaluate hK6 expression and its correlation with tumor recurrence and poor prognosis in advanced gastric cancer. Further studies will be needed to explore the possibilities whether hK6 might be used as a valuable biomarker in predicting tumor recurrence and a therapeutic target for gastric cancer by using serum samples from patients. And we also will examine the KLK6 at the mRNA level from the tissues samples next step.

## Competing interests

The authors declared that they have no competing interest.

## Authors’ contributions

XL and XY carried out pathological examination, HX and JL analyzied the data, YH drafted the manuscript. All authors read and approved the final manuscript.
